# Titanium dioxide nanoparticles perturb the blood-testis barrier via disruption of actin-based cell adhesive function

**DOI:** 10.18632/aging.203763

**Published:** 2021-12-14

**Authors:** Dong-Qi Ni, Dan-Dan Ma, Shuang-Li Hao, Wan-Xi Yang, Tamas Kovacs, Fu-Qing Tan

**Affiliations:** 1 The First Affiliated Hospital, College of Medicine, Zhejiang University, Hangzhou 310003, Zhejiang, China; 2 The Sperm Laboratory, College of Life Sciences, Zhejiang University, Hangzhou 310058, Zhejiang, China; 3 Department of Obstetrics and Gynaecology, Faculty of Medicine, University of Debrecens, Debrecen 4032, Hungary; 4 CAS Key Laboratory for Biomedical Effects of Nanomaterials and Nanosafety and CAS Center for Excellence in Nanoscience, National Center for Nanoscience and Technology, Beijing 100190, China; 5 University of Chinese Academy of Sciences, Beijing 100049, China

**Keywords:** TiO_2_-NPs, blood-testis barrier, TM-4 cell, tight junction, actin

## Abstract

As one of the most commonly used nanoparticles, titanium dioxide nanoparticles (TiO_2_-NPs) are widely used as coating reagents in cosmetics, medicine and other industries. The increasing risk of exposure to TiO_2_-NPs raises concerns about their safety. In this study, we investigated the mechanism by which TiO_2_-NPs cross the blood-testis barrier (BTB). TM-4 cells were selected as an *in vitro* Sertoli cell model of BTB. Cell viability, cell morphological changes, apoptosis, oxidative damage, and the expression levels of actin regulatory and tight junction (TJ) proteins were assessed in TM-4 cells treated with 3-nm and 24-nm TiO_2_-NPs. Cells treated with 3-nm TiO_2_-NPs exhibited increased cytotoxicity and decreased Annexin II expression, whereas cells treated with 24-nm TiO_2_-NPs exhibited increased Arp 3 and c-Src expression. Both TiO_2_-NPs induced significant oxidative stress, decreased the expression of TJ proteins (occludin, ZO-1 and claudin 5), damaged the TJ structure, and exhibited enlarged gaps between TM-4 cells. Our results indicated that both TiO_2_-NPs crossed the BTB by disrupting actin-based adhesive junctions of TM-4 cells; however, apoptosis was not observed. Our results provide new insights into how TiO_2_-NPs cross the BTB.

## INTRODUCTION

Approximately 15% of couples in Western countries are affected by infertility, among which males contribute to greater than half of the cases [1]. Over the past decades, a reduction in semen quality and count and an increasing number of infertile men have been reported [2]. Therefore, it is crucial to discover the underlying biological relevance of these changes. A previous study reported that environmental pollutants can cause infertility [3]. Nanoparticles (NPs) and other environmental pollutants cause sperm apoptosis and a reduction in sperm quality [4, 5].

Particles between 1–100 nm in size are referred to as NPs, and these new materials have been rapidly applied in various industries (*e.g.*, electronics, chemical industry, biology, medicine) and daily life [6]. Multiple engineered nanoparticles (ENPs) have been produced, including metal NPs, such as platinum, gold, and silver; metal oxide NPs, such as TiO_2_, ZnO, and Fe_2_O_3_; and carbon nanomaterials, such as C_60_ fullerenes and carbon nanotubes [7]. The high ratio of the surface area to volume of small ENPs provides these particles with unique physico-chemical properties and enhanced biological activity compared to other particles and poses a threat to the environment [8]. Mucosal, skin and organ toxicities caused by ENPs were observed in the human body after oral, skin, and respiratory exposures and injection [9]. Furthermore, ENPs can cross the blood-brain barrier (BBB) and promote the expression of proinflammatory cytokines, including IL-1*α*, TNF-*α* and NF-*κ*B [10], which may increase the risks of neurodegenerative disease and brain cancer. ENPs can also cross BTB and alter the spermatogenesis process by inducing apoptosis or necrosis of spermatocytes and Sertoli cells as well as reducing sperm motility, quality and quantity [11–13].

TiO_2_-NPs are one of the top five ENPs used in pharmaceutical products which are less cytotoxic and cytostatic to primary testicular cells than silver NPs [14]. TiO_2_-NPs are widely used in various products (*e.g.,* cosmetics, paint, sunscreens, *etc.*) and play a key role in the development of nanotechnology [15]. Nevertheless, recent papers have reported that TiO_2_-NPs can impact the male reproductive system in various ways. As endocrine disruptors, TiO_2_-NPs influence the synthesis of serum testosterone and eventually lead to reduced spermatogenesis in mice [16]. Furthermore, long-term exposure to TiO_2_-NPs could induce low fertility and inflammation in the testis, which might be associated with dysfunction of the TAM/TLR3 signaling pathway [17]. Microarray analysis revealed significant alterations in the expression of genes related to spermatogenesis and testicular function upon exposure to TiO_2_-NPs [18]. The cytotoxic and genotoxic properties of TiO_2_-NPs were assumed to affect the fertilizing potential of spermatozoa [19].

The BTB, which differs from other blood-tissue barriers, is created by coexisting TJs, basal ectoplasmic specialization (ES) and gap junctions (GJs) between adjacent Sertoli cells close to the base of the seminiferous tubule [20]. In addition, ectoplasmic specialization is a unique structure enriched with F-actin in the seminiferous epithelium that supports BTB function [21]. Sertoli cells have been isolated from humans and rodents to study functional TJ permeability, and these cells serve as an *in vivo* model to study the molecular mechanisms of TJ and BTB [22]. The mouse Sertoli cell line (TM-4) is one of the most extensively studied Sertoli cell models; these cells have been used to assess the reproductive toxicity of ENPs [23] and in BTB studies [24–26].

The underlying mechanisms of ENPs crossing BTB remain unclear. In 2012, Lan and Yang proposed the “elevator door” hypothesis [27] wherein ENPs exert inflammatory responses, which weakened BTB integrity. However, the mechanism by which microparticles that do not induce inflammatory responses cross BTB remains unknown. In our work, we studied the cytotoxic effects of 3-nm and 24-nm TiO_2_-NPs in TM-4 cells and assessed the underlying mechanisms of TiO_2_-NPs crossing BTB. We also assumed two mechanisms: (1) TiO_2_-NPs alter the expression and organization of TJ proteins, which damage the integrity of the BTB; (2) TiO_2_-NPs induce oxidative damage and apoptosis, and the loss of Sertoli cells also results in a comprised BTB.

## RESULTS

### Cytotoxicity of TiO_2_-NPs

**Figure 1:**
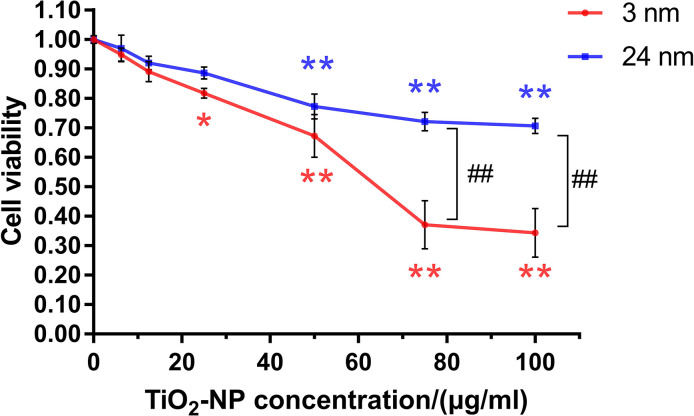
**The effects of TiO_2_-NPs on TM-4 cell viability after exposure for 24 h.** The results are expressed as the mean ± SEM. n=4, *p<0.05, **p<0.01 compared to the control; ##*p*<0.01 compared to the NPs.

Both sizes of TiO_2_-NPs showed uniform and good dispersibility (Supplementary Figure 1). TM-4 cells were incubated with two different sizes of TiO_2_-NPs at a series of concentrations (0–100 μg/ml) for 24 h, and concentration-dependent cytotoxicity of TiO_2_-NPs was observed (Figure 1). The cytotoxicity of 3-nm TiO_2_-NPs was greater than that of 24-nm TiO_2_-NPs. The median lethal concentration of 3-nm TiO_2_-NPs was ~60 μg/ml, and 24-nm TiO_2_-NPs at similar concentrations also showed a significant decrease in cell viability. Moreover, exposure of TM-4 cells to two sizes of TiO_2_-NPs at 25 μg/ml resulted in greater than 80% viability. We chose these two concentrations (30 and 60 μg/ml) of TiO_2_-NPs for further experiments based on the observed results.

### Assessment of the expression of actin regulatory proteins induced by TiO_2_-NPs

**Figure 2:**
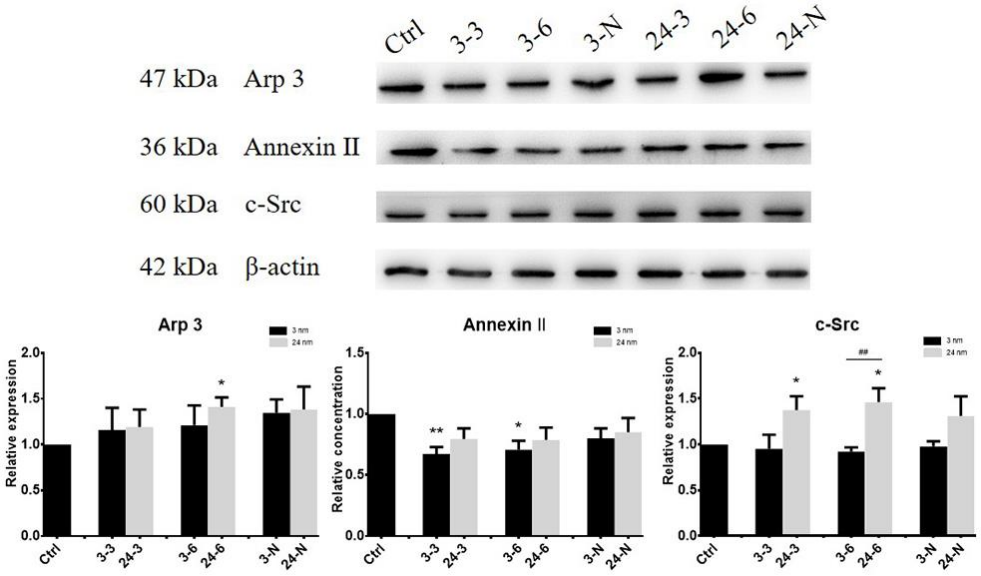
**The expression of actin regulatory proteins (Arp 3, Annexin II and c-Src) in TM-4 cells treated with two different concentrations of TiO_2_-NPs for 24 h.** “3–3”: 30 μg/ml, “3–6”: 60 μg/ml, “3-N”: TM-4 cells were treated with 5 mM NAC for 2 h and then treated with 60 μg/ml 3-nm TiO_2_-NPs for 24 h. “24–3”: 30 μg/ml, “24–6”: 60 μg/ml, “24-N”: TM-4 cells were treated with 5 mM NAC for 2 h and then treated with 60 μg/ml of 24-nm TiO_2_-NPs for 24 h. n=5, *p<0.05, **p<0.01 compared to the control; ##*p*<0.01 compared to the NPs.

The expression of actin regulatory proteins (actin-related protein 3 (Arp 3), Annexin II and a non-receptor protein tyrosine kinase (c-Src)) in TM-4 cells treated with TiO_2_-NPs at both concentrations was detected by western blotting (WB). Arp 3 helps to promote junction restructuring. Annexin II is a putative substrate of c-Src proteins, and c-Src protein is an integrated component of the BTB and apical ES in the testis [21, 28, 29]. The expression of actin regulatory proteins of TM-4 cells changed after 24 h of exposure to TiO_2_-NPs at two different concentrations (Figure 2). The expression of Arp 3 and c-Src increased after exposure to 24-nm TiO_2_-NPs, but exposure to 3-nm TiO_2_-NPs had no significant effect on expression. However, the level of Annexin II expression was slightly reduced upon exposure to 3-nm TiO_2_-NPs (30 or 60 μg/ml) for 24 h.

### Investigating reductions in BTB-associated protein expression induced by TiO_2_-NPs

**Figure 3:**
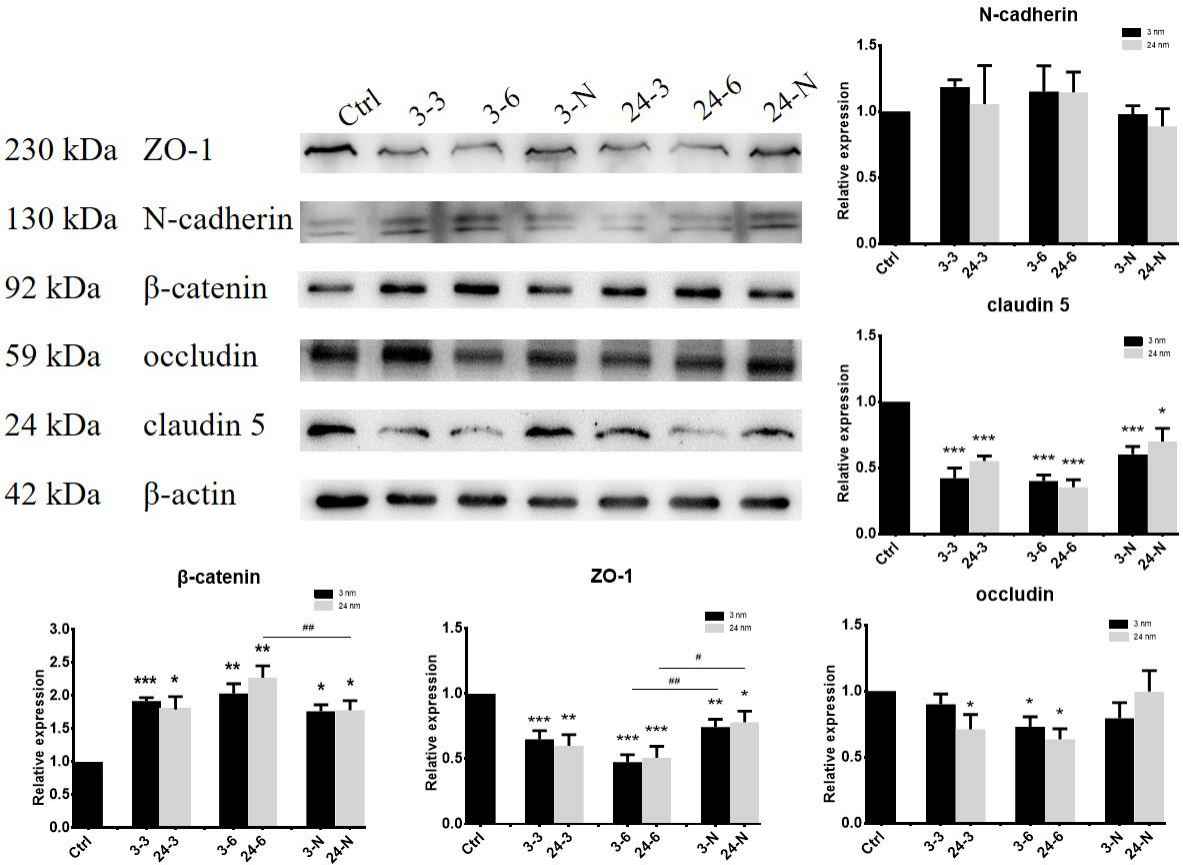
**The expression of BTB-associated proteins in TM-4 cells upon treatment with two different concentrations of TiO_2_-NPs for 24 h.** “3–3”: 30 μg/ml, “3–6”: 60 μg/ml, “3-N”: TM-4 cells were treated with 5 mM NAC for 2 h and then treated with 60 μg/ml 3-nm TiO_2_-NPs for 24 h. “24–3”: 30 μg/ml, “24–6”: 60 μg/ml, “24-N”: TM-4 cells were treated with 5 mM NAC for 2 h and then treated with 60 μg/ml of 24-nm TiO_2_-NPs for 24 h. n=5, *p<0.05, **p<0.01 compared to the control; #*p*<0.05, ##*p*<0.01 compared to the NPs.

BTB-associated proteins ZO-1 (zonula occludens-1, binds connective transmembrane proteins to the actin cytoskeleton), claudin 5 (a member of claudin family that includes integral transmembrane proteins), occludin (a tight junctional transmembrane protein), N-cadherin (a transmembrane molecule mediating calcium-dependent intercellular adhesion), and β-catenin (an adherens junction protein regulating adhesion between cells) were detected by WB after treatment with TiO_2_-NPs. ZO-1 and claudin 5 expressions were decreased, whereas β-catenin expression notably increased after treatment with two different TiO_2_-NPs (30 and 60 μg/ml). However, a decrease in occludin expression was mainly observed upon exposure to high (60 μg/ml) concentrations of TiO_2_-NPs in TM-4 cells. In addition, pretreatment with NAC partially rescued the adverse effect of TiO_2_-NPs on BTB-associated protein expression (Figure 3).

### Expanded junction gaps and F-actin remodeling in TM-4 cells induced by TiO_2_-NPs

**Figure 4:**
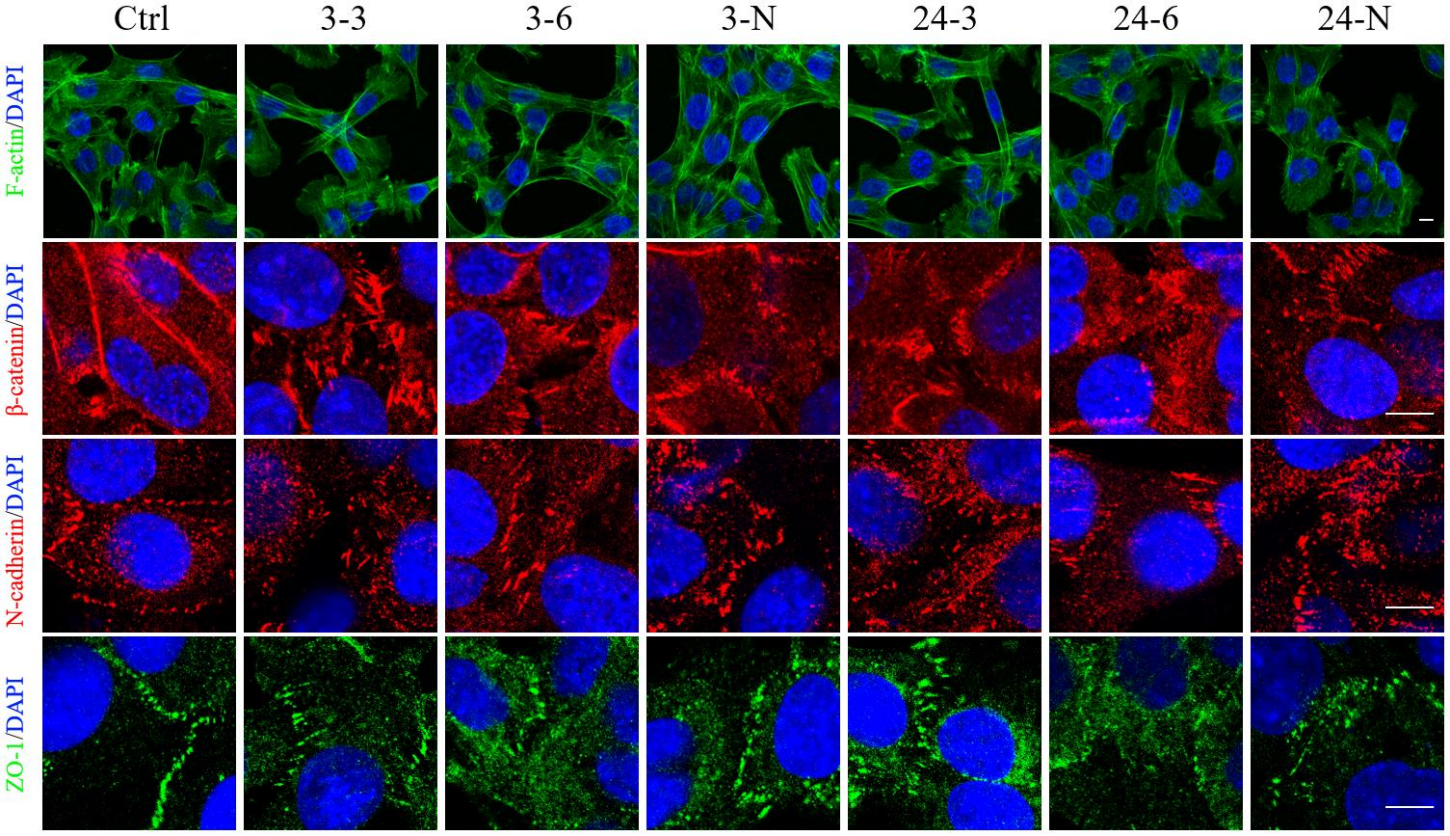
**The spatial distributions of F-actin and BTB-associated proteins in TM-4 cells treated with two different concentrations of TiO_2_-NPs for 24 h.** The distribution of intracellular microfilaments (F-actin) and the distribution of BTB-associated proteins: ZO-1 (green), N-cadherin (red), and β-catenin (red). “3–3”: 30 μg/ml, “3–6”: 60 μg/ml, “3-N”: TM-4 cells were treated with 5 mM NAC for 2 h and then treated with 60 μg/ml 3-nm TiO_2_-NPs for 24 h. “24–3”: 30 μg/ml, “24–6”: 60 μg/ml, "24-N”: TM-4 cells were treated with 5 mM NAC for 2 h and then treated with 60 μg/ml of 24-nm TiO_2_-NPs for 24 h. Scale bar=10 μm.

TM-4 cells tend to spread on the bottom of the culture plate and have typical epithelioid, protruding morphologies [30]. The edge of the cells overlapped with the long microvilli, which extended from one cell to another [31]. However, after 24 h of treatment with TiO_2_-NPs of both sizes (30 and 60 μg/ml), the number of agglomerated TM-4 cells (that did not adhere to the bottom) increased, and this effect was partially influenced by 2 h of pretreatment with NAC (Supplementary Figure 2). The density of well-attached cells after treatment with 3-nm NPs was significantly reduced compared with that noted after treatment with 24-nm NPs at the same concentrations. In normal TM-4 cells, F-actin organized to form an extensive network at the Ectoplasmic Specialization (ES) site (Figure 4). F-actin was depolymerized and then repolymerized into thick bundles in TM-4 cells treated with TiO_2_-NPs (Figure 4). Both TiO_2_-NPs induced elongated fusiform morphology of TM-4 cells (Figure 4 and Supplementary Figure 2). Additionally, NAC rescued the effect of TiO_2_-NPs on morphology (Figure 4 and Supplementary Figure 2D, 2G).

The spatial distribution of BTB-associated proteins, including β-catenin, ZO-1, and N-cadherin, was detected by immunofluorescence (IF). After treatment with TiO_2_-NPs, these three BTB-associated proteins between cells were damaged. In addition, a change in their spatial distribution was noted, and the intercellular gap was expanded significantly (Figure 4).

### TiO_2_-NPs did not induce TM-4 cell apoptosis

**Figure 5:**
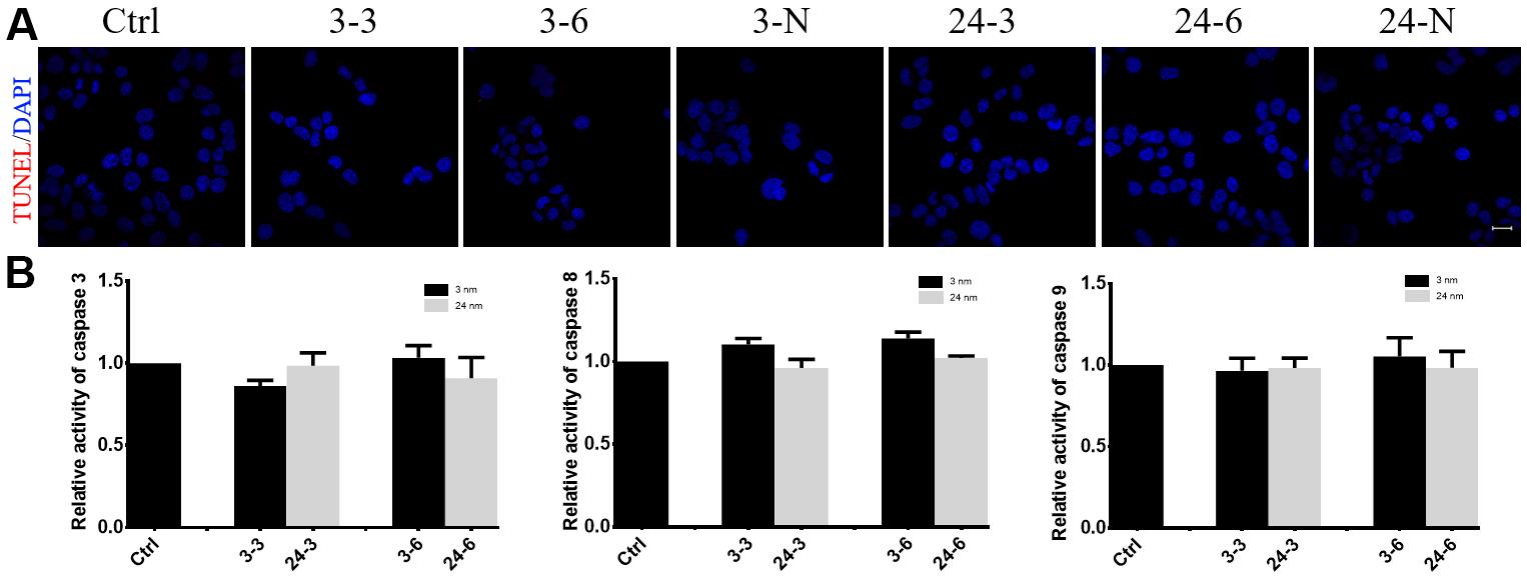
**The effect of TiO_2_-NPs on TM-4 cell apoptosis.** (**A**) TUNEL results revealed no apoptosis signals (red fluorescence) in TM-4 cells treated with TiO_2_-NPs. (**B**) Caspase 3/8/9 activity in cells treated with TiO_2_-NPs showed no obvious changes. The results were expressed as the mean ± SEM. n=4, Scale bar=20 μm.

TM-4 cell apoptosis was not observed after treatment with either concentration or size of TiO_2_-NP (Figure 5). Similar to the control, the cells treated with the NPs did not show any obvious apoptotic signals (red fluorescence) when measured using the TUNEL method (Figure 5A). Additionally, caspase 3/8/9 activity showed negligible changes in TM-4 cells after treatment with TiO_2_-NPs (Figure 5B).

### Oxidative stress in TM-4 cells treated with TiO_2_-NPs

**Figure 6:**
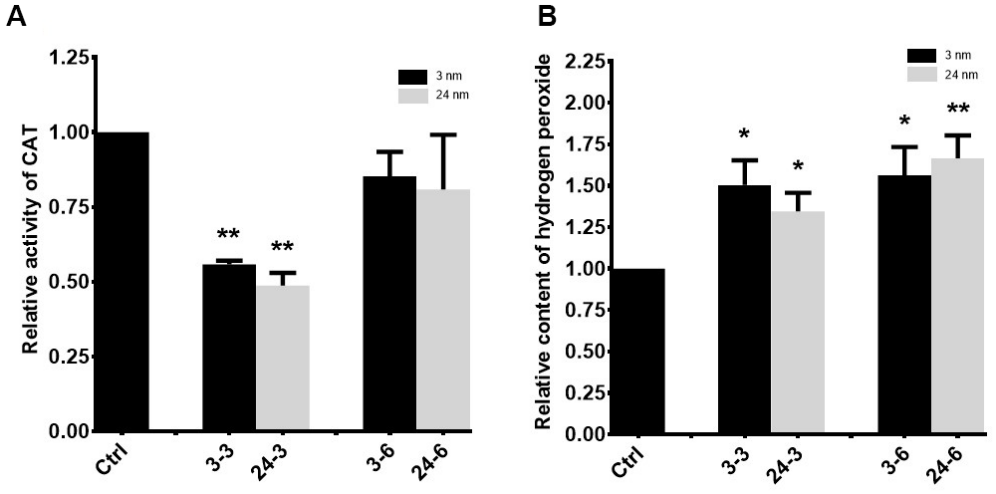
**Oxidative stress in TM-4 cells treated with TiO_2_-NPs.** (**A**) Intracellular CAT activity in TM-4 cells treated with TiO_2_-NPs for 24 h. (**B**) Intracellular hydrogen peroxide levels in TM-4 cells treated with TiO_2_-NPs for 24 h. The results are expressed as the mean ± SEM. n=7, *p<0.05, **p<0.01 compared to the control.

A significant decrease in catalase (CAT) activity was observed in TM-4 cells treated with 30 μg/ml TiO_2_-NPs for 24 h (Figure 6A), whereas no significant change in CAT activity was observed upon exposure to 60 μg/ml of either TiO_2_-NP. Hydrogen peroxide levels were enhanced in TM-4 cells treated with two different TiO_2_-NPs (Figure 6B).

## DISCUSSION

At present, *in vitro* studies of BTB have been performed using rat or human primary cultured Sertoli cells [32–34]. However, studies on the mouse BTB are rare, and most of these studies involved toxicological research on mouse primary cultured Sertoli cells [35]. The BTB consists of TJs, GPs and adhesion junctions between adjacent Sertoli cells of the testis and functions as a suitable microenvironment for spermatogenesis [36]. ENPs exert destructive effects on BTB function via disruption of Sertoli cell junctional proteins, oxidative stress, cell apoptosis, and others [32, 37, 38].

The present study showed that the toxicity of TiO_2_-NPs to TM-4 cells was concentration- and size-dependent [39–41]. The higher TiO_2_-NPs concentration showed greater toxicity to TM-4 cells. Meanwhile, the smaller sized TiO_2_-NPs presented higher toxicity to TM-4 cells. This finding might be due to the mass-specific surface area of small ENPs, which enhanced their bioactivity [42, 43]. In this study, TM-4 cells were treated with two concentrations (30 and 60 μg/ml) of TiO_2_-NPs (3 and 24 nm) and did not undergo apoptosis. Hong et al. reported that primary cultured mouse Sertoli cells treated with 15 and 30 μg/ml 5-nm TiO_2_-NPs for 24 h underwent apoptosis (32.52% and 21.02%, respectively) [35], whereas Mao et al. found that 21-nm TiO_2_ (up to 100 μg/ml for 24 h) did not reduce cell viability but induced apoptosis in TM-4 cells [41]. This finding indicates that the cytotoxic effects of TiO_2_ on mouse Sertoli cells is based on the sizes of TiO_2_-NPs and cell types.

The BTB is a strong structure composed of Sertoli cells, vascular endothelial basement membrane, connective tissue, and compact tubule basement membrane. The BTB prevents cytotoxic substances from entering the seminiferous tubules to avoid the influence of foreign substances on spermatogenic cells. In normal Sertoli cells from rats and humans, F-actin was distributed in organized bundles in the cell membrane [20, 28, 44]. In this study, F-actin was transformed from a well-organized and fine structure to a thick polymerized bundle-like structure after exposure to TiO_2_-NPs. This change is due to the effects of TiO_2_-NPs on the expression of actin regulatory proteins, including Arp 3, Annexin II and c-Src, which are all involved in the depolymerization and polymerization of F-actin [21, 36, 37]. The reorganization of F-actin caused changes in the spatial organization of actin in ES, which affects the overall structure of the tight junction.

TJs are the main structure of the BTB and include claudins, occludin, ZO proteins among others [45]. As the first TJ transmembrane protein discovered [46], occludin is responsible for regulating the barrier properties of TJs [47]. ZO-1 belongs to the family of membrane-associated guanylate kinase proteins [20] and provides a linkage between the actin cytoskeleton and TJ proteins, such as occludin, JAMs and claudins [48]. Claudin 5, a member of the claudin family, which comprise the major component of TJ strands, is an integral membrane protein that shares the transmembrane domains of occludin [49]. *Claudin 5* knockout caused spermatogenic cells to fall off into the seminiferous tubule lumen [50]. N-cadherin and β-catenin are adhesion protein complexes that assemble at the Sertoli cell-cell interface to form cell adhesion [28]. Our results demonstrated that two different sizes of TiO_2_-NPs could decrease the levels of ZO-1, occludin and claudin 5 in TM-4 cells and perturb the spatial positions of ZO-1, N-cadherin and claudin 5. In contrast, the gaps were increased, indicating that TiO_2_-NPs expanded TJ gaps.

Hong et al. reported that 5-nm TiO_2_-NPs reduced CAT activity in mouse Sertoli cells and induced oxidative damage in these Sertoli cells [35]. Our results revealed a similar oxidative stress response. Moreover, pretreatment with NAC (antioxidant) partly rescued the decrease in cell-cell junction proteins and the morphology of TM-4 cells treated with TiO_2_-NPs according to the western blot and immunofluorescence results. These findings indicate that oxidative stress is one of the factors that disrupts the connection between TM-4 cells and TiO_2_-NPs.

**Figure 7:**
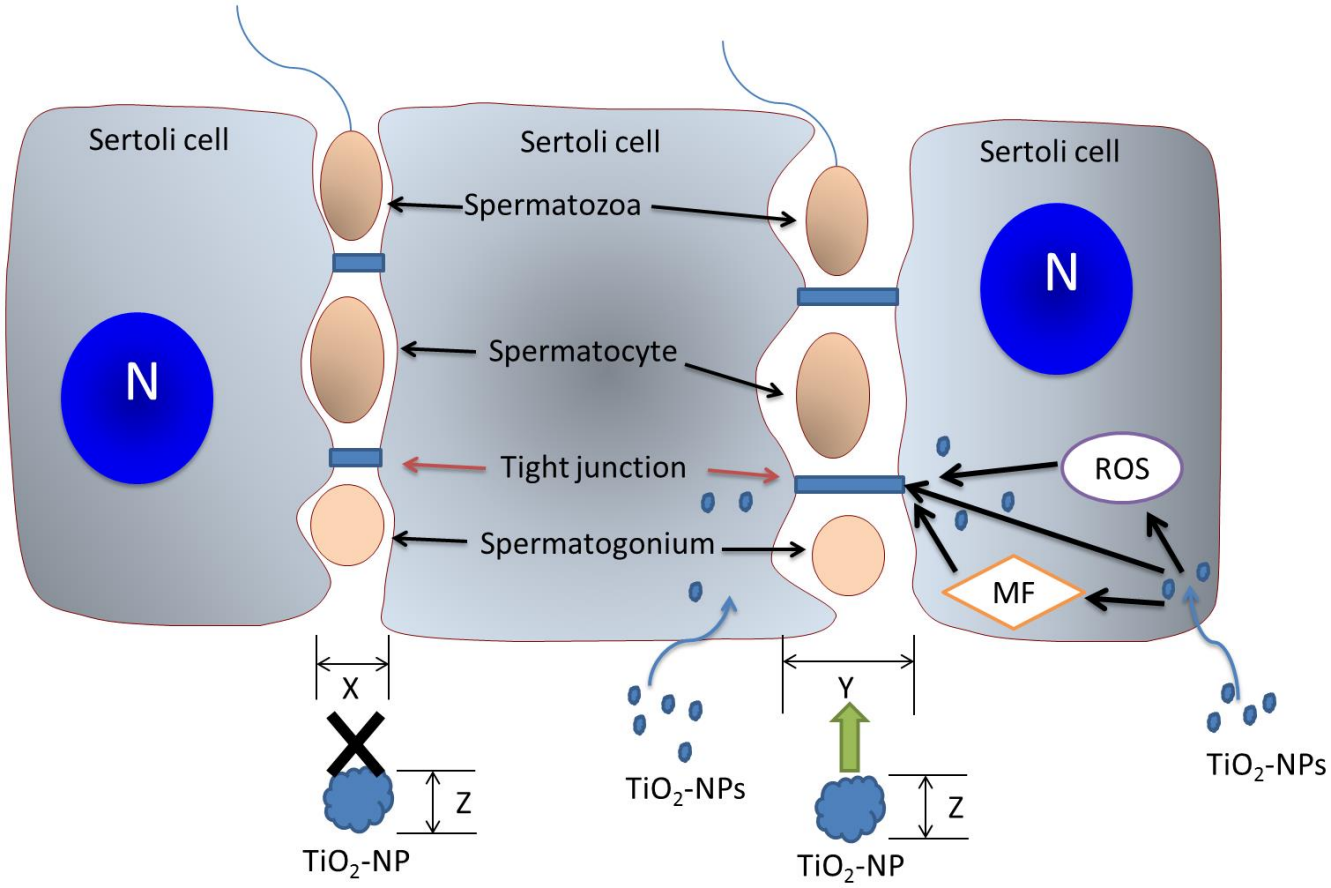
**The mechanism of action of TiO_2_-NPs on BTB.** TiO_2_-NPs damage the BTB via three mechanisms. TiO_2_-NPs interfere with BTB-associated proteins, induce cytoskeletal reorganization, and trigger oxidative stress to perturb TJ proteins. The BTB gap (size X) is expanded (size Y) by TiO_2_-NPs, and TiO_2_-NPs (particle size Z<Y) with particle sizes less than Y pass directly through the BTB gap and subsequently affect spermatogenesis. N: nucleus; MF: microfilament.

Taken together, TiO_2_-NPs damage the spatial structure of BTB-associated proteins and reorganize the cytoskeletal structure. We further confirmed that TiO_2_-NPs induce oxidative stress to perturb the content of BTB-associated and actin regulatory proteins, thus affecting the structure of TJs and the cytoskeleton (Figure 7). Our study showed that TiO_2_-NPs of both sizes could expand the BTB gaps to allow small TiO_2_-NPs to cross the BTB and affect spermatogenesis.

## MATERIALS AND METHODS

### Chemicals

TiO_2_-NPs (3 nm and 24 nm) were purchased from JCNANO (Nanjin, China). The ultrastructural characteristics of TiO_2_-NPs are shown in Supplementary Figure 1.

### Cell line

TM-4 cells from ATCC were maintained at 37° C with 5% CO_2_ in DMEM/F12 (Genom, China) supplemented with 1.25% fetal bovine serum, 2.5% horse serum (Zhejiang Tianhang Biotechnology, China), and 100 U/ml penicillin/streptomycin. When 50% cell confluence was achieved, different concentrations of TiO_2_-NPs (3 nm and 24 nm) were added, and the cells were incubated for 24 h.

### Cell viability assay

TM-4 cells in 96-well plates were treated with various concentrations (0, 6.25, 12.5, 25, 50, 75 and 100 μg/mL) of TiO_2_-NPs (3 nm and 24 nm) for 24 h. Then, TM-4 cell viability was determined using the Cell Counting Kit-8 (Beyotime, China). OD450 values were determined using the multiplate reader Multiskan FC (Thermo).

### Western blotting

TM-4 cells were lysed in RIPA buffer (Beyotime, China) with 1% protease inhibitor (Beyotime, China). Samples were separated by electrophoresis and transferred to PVDF membranes (Millipore, USA). Then, the samples were incubated with primary antibodies (Table 1) at 4° C overnight and incubated with secondary HRP-conjugated antibodies the next day for 1 h at room temperature (RT). The images were detected using an enhanced chemiluminescent kit (Beyotime, China). The bands were quantified using ImageJ.

**Table 1:** Antibodies used for different experiments.

**Antibody**	**Host species**	**Vendor**	**Catalog number**	**Application(s)/dilution(s)**
Anti-β-actin rabbit polyclonal antibody	rabbit	Sangon Biotech	D110001	WB(1:2000)
ZO-1 Antibody	rabbit	Proteintech Group	21773-1-AP	WB(1:500)
c-Src(B-12)	mouse	Santa Cruz Biotechnology	sc-8056	WB(1:200)
Anti-ACTR3 rabbit polyclonal antibody	rabbit	Sangon Biotech	D154021	WB(1:1000)
Annexin II(H-50)	rabbit	Santa Cruz Biotechnology	sc-9061	WB(1:200)
Occludin Antibody	rabbit	Proteintech Group	13409-1-AP	WB(1:500), IF(1:50)
Anti-claudin 5	rabbit	Abcam	ab15106	WB(1:1000), IF(1:200)
β-catenin mouse monoclonal antibody	mouse	Beyotime	AF0069	WB(1:600), IF(1:200)
N-Cadherin rabbit polyclonal antibody	rabbit	Beyotime	AF0243	WB(1:500), IF(1:500)
Actin-Tracker Green	rabbit	Beyotime	C1033	IF(1:100)

WB, western blotting; IF, immunofluorescence analysis.

### Immunofluorescence

TM-4 cells were fixed in ethanol for 10 min (ZO-1) or 4% paraformaldehyde for 15 min (N-cadherin, β-catenin and F-actin). After washing three times and blocking with 5% BSA for 1 h, TM-4 cells were incubated with Actin-Tracker Green or ZO-1 antibody for 1 h and N-cadherin or β-catenin antibody overnight at 4° C followed by incubation with Alexa Fluor 555/488 donkey-anti-rat/mouse IgG (Beyotime, China). Nuclei were stained blue with DAPI (Beyotime, China). After mounting with Antifade Mounting Medium (Beyotime, China), the samples were detected using a confocal laser-scanning microscope (CLSM 510) (Carl Zeiss, Germany).

### Cell apoptosis assay

A Caspase 3/8/9 Activity Assay Kit (Beyotime, China) was used to assess caspase 3/8/9 activity. TM-4 cells in 6-well plates were treated with TiO_2_-NPs for 24 h and then digested with trypsin for 2 min. Total proteins were extracted using RIPA buffer (Beyotime, China). The supernatants were collected by centrifugation at 13,000 rpm for 15 min at 4° C. The test buffer, the samples, and Ac-DEVD/IETD/LEHD-pNA (2 mM) were added to 96-well plates and incubated at 37° C for 2–4 h or overnight. Then, OD405 was measured. Protein concentrations were measured using the Bradford method to calculate the units of caspase 3/8/9 activity per sample based on the units of weight protein.

For the TUNEL apoptosis assay, TM-4 cells were fixed in 4% PFA for 30 min and permeabilized in 0.3% Triton X-100 for 5 min. Then, samples were incubated with TUNEL test solution (Beyotime, China) for 1 h at 37° C. Nuclei were stained blue with DAPI (Beyotime, China). After mounting with Antifade Mounting Medium (Beyotime, China), the samples were detected using a confocal laser-scanning microscope (CLSM 510) (Carl Zeiss, Germany).

### Determination of hydrogen peroxide levels and catalase (CAT) activity

Intracellular hydrogen peroxide levels and intracellular CAT activity were determined using a hydrogen peroxide assay kit (Beyotime, China) and catalase assay kit (Beyotime, China), respectively.

Briefly, for the determination of intracellular hydrogen peroxide, a 50-μl sample was added to a 96-well plate. Then, 100 μl hydrogen peroxide detection reagent was added and mixed well. Then, the mixture was incubated at RT for 30 minutes, and the OD546 was immediately measured. The concentration of intracellular hydrogen peroxide was calculated based on the standard curve.

For the determination of intracellular CAT, 2 μl sample, 38 μl catalase test buffer were added into the centrifuge tube, then with another 10 μl of 250 mM hydrogen peroxide solution, the mixture was incubated at 25° C for 1–5 min. Then, 450 μl catalase reaction stop solution was added to stop the reaction. Ten microliters of the above mixture was added to 40 μl catalase assay buffer. After mixing well, the mixture was added to color working solution in a 96-well plate. The cells were incubated at 25° C for at least 15 min, and then the OD520 was measured. Intracellular CAT activity was calculated based on the standard curve of CAT.

### Data analysis

Graphical analysis was performed using GraphPad Prism 7. The data were expressed as the mean ± SEM. Student’s t-test was used to study statistical significance between two groups. One-way ANOVA was used to study statistical significance between multiple groups.

## Supplementary Material

Supplementary Figures
