# Supplementary material for: Titanium dioxide nanoparticles perturb the blood-testis barrier via disruption of actin-based cell adhesive function
Source: Aging (Albany NY). 2021 Dec 14;13(23):25440–52. doi: 10.18632/aging.203763 (PMC8714145; doi:10.18632/aging.203763)
Supplement: Supplementary Figures [file aging-13-203763-s001.pdf]

SUPPLEMENTARY FIGURES

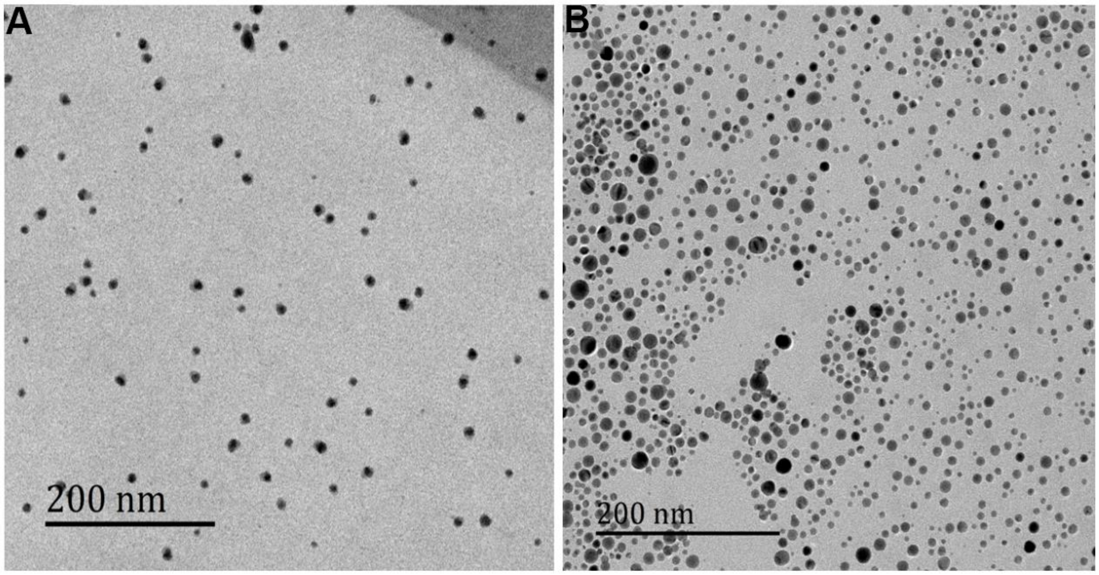

Supplementary Figure 1. TiO_2-NP transmission electron microscopy. (**A**) 3-nm TiO_2-NPs and (**B**) 24-nm TiO_2-NPs (transmission electron micrographs were provided by Nanjing Jicang Nano Technology).

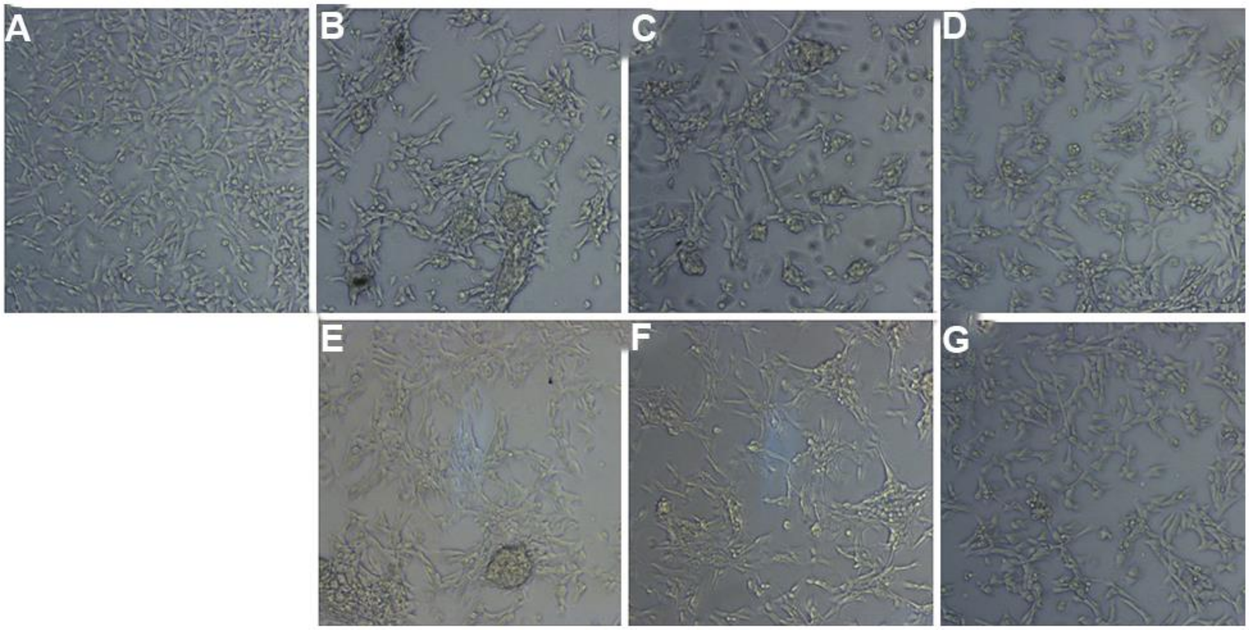

Supplementary Figure 2. Morphological changes in TM-4 cells treated with TiO_2-NPs. (**A**) Control group, (**B**) 30 μg/ml 3-nm TiO_2-NP treatment for 24 h, (**C**) 60 μg/ml 3-nm TiO_2-NP treatment for 24 h, (**D**) 60 μg/ml 3-nm TiO_2-NP treatment for 24 h prior to treatment with NAC for 2 h, (**E**) 30 μg/ml 24-nm TiO_2-NP for 24 h, (**F**) 60 μg/ml 24-nm TiO_2-NP for 24 h, and (**G**) 60 μg/ml 24-nm TiO_2-NP treatment for 24 h prior to treatment with NAC for 2 h. ×100.
